# Implementation and results of an integrated data quality assurance protocol in a randomized controlled trial in Uttar Pradesh, India

**DOI:** 10.1186/s13063-017-2159-1

**Published:** 2017-09-07

**Authors:** Jonathon D. Gass, Anamika Misra, Mahendra Nath Singh Yadav, Fatima Sana, Chetna Singh, Anup Mankar, Brandon J. Neal, Jennifer Fisher-Bowman, Jenny Maisonneuve, Megan Marx Delaney, Krishan Kumar, Vinay Pratap Singh, Narender Sharma, Atul Gawande, Katherine Semrau, Lisa R. Hirschhorn

**Affiliations:** 1 Ariadne Labs of the Brigham & Women’s Hospital and Harvard T.H. Chan School of Public Health, Boston, MA USA; 2 Population Services International, New Delhi, India; 3 Ariadne Labs, Harvard T.H. Chan School of Public Health, Brigham & Women’s Hospital, Northwestern University Feinberg School of Medicine, Arthur J. Rubloff Building 420 East Superior Street, Chicago, 60611 Illinois USA

**Keywords:** Data Quality Assurance (DQA), Safe Childbirth Checklist (SCC), Maternal morbidity, Maternal and perinatal mortality, Data feedback, Supportive supervision, Patient-reported outcomes, Uttar Pradesh, India, Data accuracy, Randomized control trial (RCT)

## Abstract

**Background:**

There are few published standards or methodological guidelines for integrating Data Quality Assurance (DQA) protocols into large-scale health systems research trials, especially in resource-limited settings. The BetterBirth Trial is a matched-pair, cluster-randomized controlled trial (RCT) of the BetterBirth Program, which seeks to improve quality of facility-based deliveries and reduce 7-day maternal and neonatal mortality and maternal morbidity in Uttar Pradesh, India. In the trial, over 6300 deliveries were observed and over 153,000 mother-baby pairs across 120 study sites were followed to assess health outcomes. We designed and implemented a robust and integrated DQA system to sustain high-quality data throughout the trial.

**Methods:**

We designed the Data Quality Monitoring and Improvement System (DQMIS) to reinforce six dimensions of data quality: accuracy, reliability, timeliness, completeness, precision, and integrity. The DQMIS was comprised of five functional components: 1) a monitoring and evaluation team to support the system; 2) a DQA protocol, including data collection audits and targets, rapid data feedback, and supportive supervision; 3) training; 4) standard operating procedures for data collection; and 5) an electronic data collection and reporting system. Routine audits by supervisors included double data entry, simultaneous delivery observations, and review of recorded calls to patients. Data feedback reports identified errors automatically, facilitating supportive supervision through a continuous quality improvement model.

**Results:**

The five functional components of the DQMIS successfully reinforced data reliability, timeliness, completeness, precision, and integrity. The DQMIS also resulted in 98.33% accuracy across all data collection activities in the trial. All data collection activities demonstrated improvement in accuracy throughout implementation. Data collectors demonstrated a statistically significant (*p* = 0.0004) increase in accuracy throughout consecutive audits. The DQMIS was successful, despite an increase from 20 to 130 data collectors.

**Conclusions:**

In the absence of widely disseminated data quality methods and standards for large RCT interventions in limited-resource settings, we developed an integrated DQA system, combining auditing, rapid data feedback, and supportive supervision, which ensured high-quality data and could serve as a model for future health systems research trials. Future efforts should focus on standardization of DQA processes for health systems research.

**Trial Registration:**

ClinicalTrials.gov identifier, NCT02148952. Registered on 13 February 2014.

## Background

There are no widely accepted universal standards for data quality in health systems research, despite several articles and reports emphasizing their importance [1–10]. While there are known methods for assessing data quality in patient registries and health information systems, there are few published methodological guidelines for integrating Data Quality Assurance (DQA) protocols into large-scale health systems research trials, especially in resource-limited settings [5, 9, 11–14]. High-quality data are crucial in health systems research as scientific recommendations based on those data have implications for policy and practice [5, 8].

Error rates in clinical trials have been described in the literature ranging from 2.8% to 26.9% across multiple studies [15–20]. There are no minimally acceptable data-quality standards included in US Federal guidelines for clinical research; therefore, researchers establish their own acceptable error rates and measurement methods [10]. Onsite monitoring of clinical trial sites and database audits occur; however, published systematic approaches to field verification of data quality during trial implementation are rare, and their absence limits opportunities to remediate data-quality issues in real time [14, 21, 22]. Clinical trials often require multiple data collection activities, all subject to different sources of error; therefore, DQA activities often must target multiple dimensions of quality [14, 16, 23]. DQA methods must address all possible sources of error in an integrated, systematic, and supportive manner to promote continuous data quality improvement throughout implementation [24, 25].

The BetterBirth Trial is a matched-pair, cluster-randomized controlled trial (RCT) of the BetterBirth Program, which uses coaching-based implementation of the World Health Organization (WHO) Safe Childbirth Checklist to improve quality of facility-based deliveries in Uttar Pradesh, India, and to reduce 7-day maternal and neonatal morbidity and mortality [26]. This complex and large-scale trial includes three sources of data: patient registry, delivery observation, and post-delivery patient-reported outcomes data. In the trial, over 6300 deliveries were observed, and over 153,000 mother-baby pairs across 120 study sites were followed to assess health outcomes [27]. We designed and implemented the Data Quality Monitoring and Improvement System (DQMIS), a robust, multi-component, and integrated DQA mechanism, to ensure high-quality data throughout study implementation. This study aimed to evaluate the DQMIS and its effectiveness for ensuring data quality. In the absence of published approaches to field verification of data quality during trials, here we report the implementation components and results of an integrated DQA system.

## Methods

### Data collection activities in the BetterBirth Trial

The trial included five data collection activities related to the three sources of data. 1) Essential birth practices performed by birth attendants during deliveries were observed and recorded by facility-based observers. Following observations, 2) the observation data recorded on paper forms were transferred to the electronic data entry app. 3) Patient data, sourced from paper-based facility registers, were extracted by facility-based data collectors and entered into a paper-based study register. Following data extraction, 4) patient data were transferred to the electronic data entry app by facility-based data collectors. Finally, 5) call center staff contacted patients to assess maternal and neonatal mortality and seven maternal morbidities using a standardized questionnaire [27] and entered these data directly into the electronic data collection app.

### Design of the DQMIS

We designed the DQMIS to reinforce six dimensions of data quality [28] (Table 1). The DQMIS comprised of five complementary functional components, including: 1) a monitoring and evaluation (M&E) team to support data management and quality; 2) a DQA protocol, including data collection audits and targets, rapid data feedback, and supportive supervision; 3) training on data quality; 4) standard operating procedures (SOPs) for data collection; and 5) an electronic data collection and reporting system (Table 2).

**Table 1:** Operational definitions for six dimensions of data quality, adapted from Brown W, et al. [28]

Accuracy	Data are correct and reflect the truth
Reliability	Data are consistently collected and entered in a standard way across data collectors
Timeliness	Data are current due to routine data entry and available for near real-time reporting
Completeness	There are no missing essential data elements
Precision	Data have necessary detail to address research questions and management requirements
Integrity	Data are secure and protected from bias or manipulation

**Table 2:** Functional components of the DQMIS and corresponding dimensions of data quality

	Dimensions of data quality					
Functional components of the DQMIS	Accuracy	Reliability	Timeliness	Completeness	Precision	Integrity
M&E team to support data management and quality	X	X	X	X	X	X
SOPs and tools for data collection	X	X	X		X	X
Training for data quality	X	X	X			X
Electronic data collection and reporting system	X	X	X	X	X	X
DQA protocol, including data collection audits, rapid data feedback, and supportive supervision	X	X				

*DQA* Data Quality Assurance, *DQMIS* Data Quality Monitoring and Improvement System, *M&E* Monitoring and evaluation

#### Functional components of the DQMIS

##### Monitoring and evaluation (M&E) team support for data management and quality

Two M&E staff managed operations of the DQMIS across all data collection activities and provided technical assistance and capacity development to supervisory staff in the field. The M&E team was responsible for oversight of all functional components of the DQMIS, including the DQA protocol, organizing trainings, developing and revising SOPs as needed, and providing technical assistance on data collection and report interpretation. The M&E reinforced all six dimensions of data quality throughout the trial.

##### Standard operating procedures (SOPs) for data collection

Tools were designed and SOPs for each data collection activity were defined prior to study start. All data collection tools were programmed into the electronic component of the data collection system to facilitate automated and scalable data quality monitoring. SOPs included frequency, method, and technique for each data collection activity.

##### Training

All data collectors and supervisors participated in an 8-day orientation training program focused on implementation of SOPs, data-collection tools, the electronic data collection apps, and reporting system. As a part of this orientation, a 1-day training focused on the functional components of the DQA protocol. Additionally, data collectors engaged in active learning by visiting facilities to learn study implementation processes in the field. Subsequent staff-wide and staff-specific refresher trainings were delivered throughout implementation of the trial.

##### Electronic data collection and reporting system

We developed a data collection and reporting system to centralize data management for the trial. The system included front-end smartphone and tablet-based electronic data collection applications (based off Dimagi’s open-source CommCare platform) for each data collection tool, a secure cloud-based server for data storage and integrity, and a reporting portal for study operations, including data quality. The reporting system produced data quality reports using pre-defined algorithms and data visualizations to facilitate near real-time feedback on accuracy of trial data.

##### DQA protocol, including audits, real-time data feedback, supportive supervision

We designed a standardized DQA protocol as an integrated component of the trial to continuously assess and improve data accuracy and reliability throughout implementation. Supervisors performed audits on data collectors to address quality of the five data collection activities. Audits targeted accuracy of data entry, delivery observations, and patient-reported outcomes ascertained by the call center. The auditing process, unique for each data collection activity, required perfect accuracy on a sample of data collected by each data collector in a phased approach. Following orientation, each data collector began an intensive phase of auditing lasting 6 weeks (or longer in case of any difficulty achieving targets). After achieving performance targets of the intensive phase, data collectors graduated into a maintenance phase, with audits repeating every 3 months. No a priori decisions were made regarding the proportion of data in each data collection activity that would be assessed for quality; rather, the data collector’s ability to achieve set targets determined the proportion of data within each data collection activity that was checked for accuracy. Perfect accuracy was required for each performance target; any errors required that the audit be repeated from the beginning (Table 3).

**Table 3:** Data sources and audit methods

Data source	Data collection process	Audit process	Intensive phase target and duration	Monitoring phase target and frequency
*Accuracy of observation of birth attendant practices*				
Birth practices performed by birth attendant during deliveries	Direct observation of deliveries with data entry into paper-based checklist by facility-based observers	Simultaneous observation by supervisor	100% accuracy on three consecutive simultaneous observations of each of four observation points (OPs); first 4 weeks after hire	100% accuracy on three consecutive simultaneous observations at each OP (OP1, OP2, OP3, OP4); every 3 months
*Accuracy of data entry*				
Observation checklist of birth attendant practices	Data entry of paper-based delivery observation data into electronic data-entry app by facility-based observers	Double data entry by supervisors	100% accuracy on two sets of 10 sequentially entered forms; first 4 weeks after hire	100% accuracy on one set of 10 sequentially entered forms; every 3 months
Facility registers	Extraction of patient data from paper-based facility registers into paper-based study register by facility-based data collectors	Cross verification of extracted data with facility-based register data by supervisors	No intensive phase	100% accuracy on a consecutive set of 10 patients’ extracted register information; monthly
Study register	Data entry of patient data from paper-based study register to electronic data entry app by facility-based data collectors	Double data entry by supervisors	No intensive phase	100% accuracy on a consecutive set of 10 patients’ register information; monthly
*Accuracy of patient-reported outcomes*				
Patient-reported outcomes	Call center staff contact patients to assess maternal and neonatal mortality and seven maternal morbidities using standardized data collection tool	Recorded call review and double data entry into electronic data entry app by supervisor	100% accuracy on four sets of 10 sequentially reviewed calls; first 4 weeks after hire	100% accuracy on four sets of 10 sequentially reviewed calls; every 3 months

**Fig. 1:**
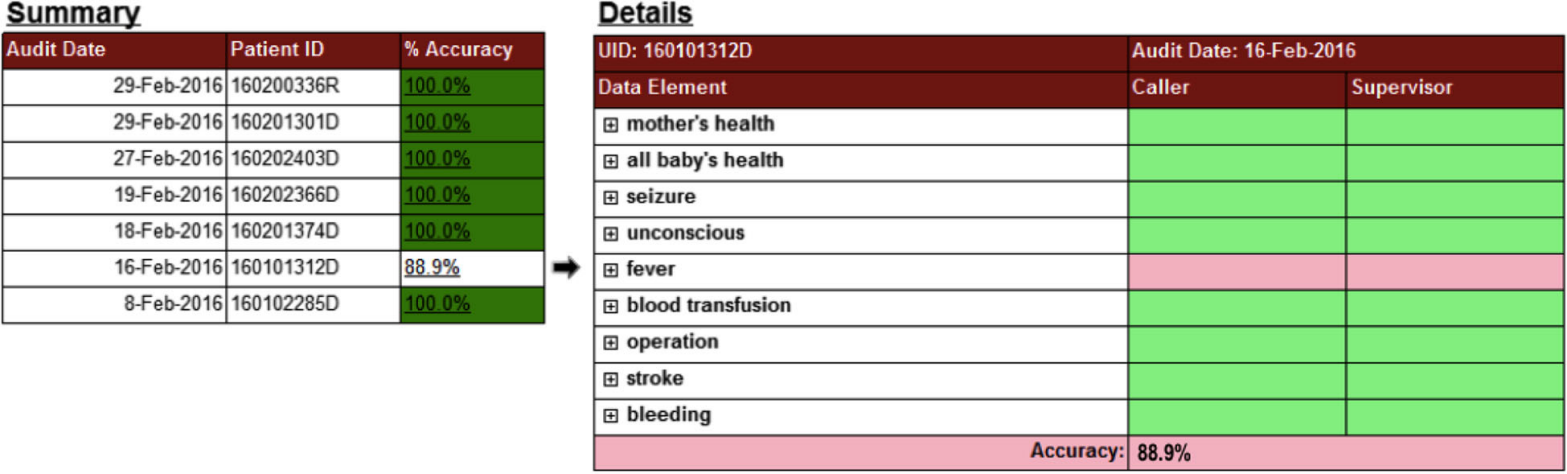
Data quality accuracy report for patient-reported outcomes

The DQA protocol was supported by rapid, timely, and automatic data quality feedback. Data quality reports were designed to inform supervisors and study management staff of audit results at the level of data collector, including accuracy rates, DQA phase, error trends, target achievement, and data entry delay. Additionally, reports designed for study management presented aggregated accuracy rates and error trends across data collectors. Reports were available within 24 h of audits and accessed via smartphone and tablet. In observance of blinding rules related to observation and outcomes data for certain staff, reports displayed accuracy in green and errors in red, rather than the actual data (Fig. 1).

In addition, we designed a supportive supervision model to facilitate data accuracy and reliability (quality improvement (QI)) across all data collection activities. Experienced supervisors were assigned to support specific data collectors in order to build trust and rapport. Utilizing the reporting system, supervisors reviewed audit results on a continual basis to identify target accomplishment and occurrence of errors. Thereafter, immediate onsite support was provided to data collectors. Success was celebrated, and challenges were addressed in a supportive manner. First, supervisors shared accuracy reports with staff to address challenges. Second, sources of error were discussed, whether they were related to data entry, interpretation, or technical aspects of the app. Finally, supervisors and data collectors together devised strategic plans to improve accuracy, which included refresher training, one-on-one support, and peer-to-peer mentorship. The M&E team provided ongoing support to supervisors in this process.

### Data analysis

Descriptive statistics were calculated for accuracy results, including proportion of forms evaluated for accuracy, overall accuracy, and accuracy by data collection activity. The proportion of forms evaluated for accuracy was calculated as the number of forms audited out of the total number of forms collected over the same time period (7 November 2014 to 6 September 2016). The percent accuracy was calculated for all forms audited. A form was considered accurate if all questions were consistent between both entries of the form. A form was considered inaccurate if it contained one or more errors. The percent accuracy for forms for each activity was plotted over time by month and assessed for trends. The relative risk of accuracy for each data collection activity by each consecutive form audited was calculated using relative risk regression, clustered by data collector [29, 30]. All statistical analyses were performed using SAS 9.4®.

## Results

Data collection staff gradually increased as the volume of data increased over the course of the trial. At their maximum, data collection staff included 32 facility-based observers (26 data collectors, six supervisors), 116 facility-based field workers (78 data collectors, 38 supervisors), and 33 call center staff (26 callers, 6 supervisors, 1 manager).

### Completeness, precision, and integrity

These three dimensions of data quality were primarily guaranteed through the back-end design of the data collection and reporting system. All electronic data collection apps included required fields and skip patterns to prevent missing values upon data entry, guaranteeing completeness of all datasets. Data precision was protected through data definitions and field restrictions in the electronic data collection system. The secure cloud-based server certified the integrity of data by preventing data manipulation by any staff.

### Timeliness and reliability

Timeliness of data was reinforced by the SOPs for data collection and by routine staff trainings, which emphasized that each data collector enter data from paper-based forms to electronic apps as soon as possible after data collection. For the two data collection activities for which primary data collection was paper-based (data entry of observation checklist, and data entry of patient data into the study register), the mean duration until electronic entry was 0.46 and 2.14 days, respectively. Reliability of data was accomplished through all five functional components of the DQMIS, collectively ensuring consistency in data collection across data collectors.

### Proportion and accuracy of trial data audited

Among the five data collection activities, the proportion of forms (case-level data) audited ranged from 2.17% to 39.32%. The DQA protocol resulted in a high overall rate of accuracy across all data collection activities in the trial, with accuracy of each data collection activity ranging from 91.77% to 99.51% (Table 4).

**Table 4:** Proportion and accuracy of trial data audited (7 Nov 2014 to 6 Sept 2016)

Data collection activity	Total forms (*N*)	Forms audited (*n*)	Proportion of total forms audited (%)	Forms audited with total accuracy (*n*)	Proportion of forms audited with total accuracy (%)
Observation of birth attendant practices					
OP1: On admission	4886	436	8.92%	431	98.85%
OP2: Just before delivery	5000	479	9.58%	445	92.90%
OP3: Within 1 min after delivery	4998	461	9.22%	454	98.48%
OP4: Within 1 h after delivery	4854	465	9.58%	451	96.99%
Data entry of observation checklist	5933	2333	39.32%	2141	91.77%
Data extraction of patient data from facility register to study register	136,057	10,341	7.60%	10,290	99.51%
Data entry of patient data from study register to app	136,057	8221	6.04%	8155	99.20%
Patient-reported outcomes	110,475	2400	2.17%	2350	97.92%
Overall	408,260	25,136	6.16%	24,717	98.33%

*OP* observation point

### Accuracy of trial data over time

**Fig. 2:**
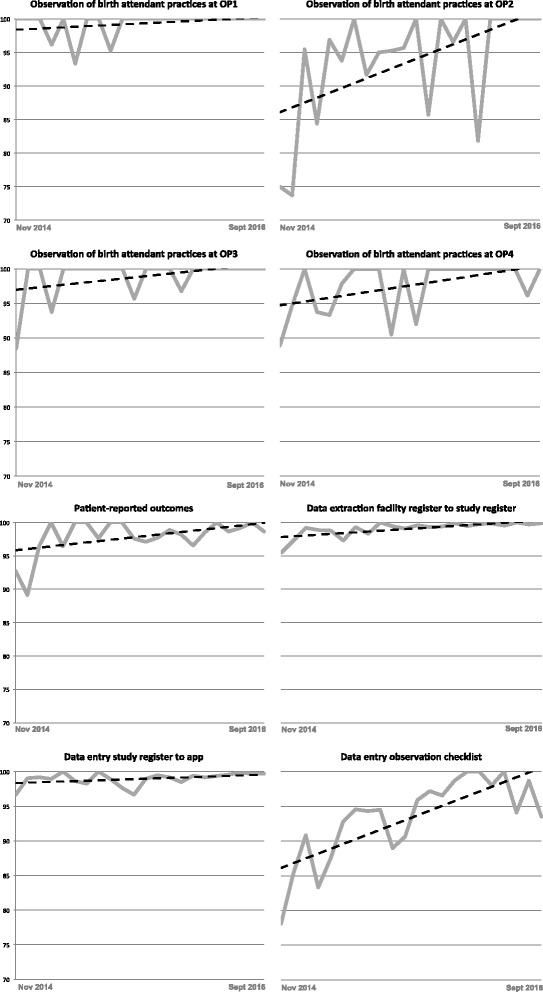
Accuracy rate and trend of each data collection activity by month (7 Nov 2014 to 6 Sept 2016). *OP* observation point

All data collection activities demonstrated an upward trend in accuracy improvement throughout implementation. For example, monthly accuracy of observation of birth attendant practices at observation point (OP)2 increased from 73.68% to 100% (Fig. 2). The accuracy of each question in all data collection activities was also analyzed. Over time, question-level accuracy never decreased. In most instances, question-level accuracy remained high throughout and, in several instances, question-level accuracy improved over time.

### Accuracy of data collectors over time

Data collector accuracy remained high from the first audit through all consecutive audits. A small but significant increase in accuracy was achieved throughout consecutive audits for three of the data collection activities and for three of the four OPs. For the other data collection activities, there was no significant change in data collector accuracy as it remained high throughout the trial. In no case did accuracy decrease among data collectors throughout consecutive auditing (Table 5).

**Table 5:** Unadjusted trend in accuracy of data collectors over time

Data collection activity	RR (95% CI)	*p* value
Observation of birth-attendant practices		
OP1: On admission	1.0003 (0.9995-1.0011)	0.4140
OP2: Just before delivery	1.0043 (1.0006-1.0081)	0.0242
OP3: Within 1 min after delivery	1.0015 (1.0003-1.0027)	0.0119
OP4: Within 1 h after delivery	1.0019 (0.9999-1.0039)	0.0679
Data entry of observation checklist	1.0006 (1.0000-1.0012)	0.0366
Data extraction of patient data from facility register to study register	1.0000 (1.0000-1.0001)	0.7218
Data entry of patient data from study register to app	1.0000 (1.0000-1.0001)	0.0473
Patient-reported outcomes	1.0003 (1.0000-1.0005)	0.0304
Total combined trend in accuracy	1.0001 (1.0000-1.0002)	0.0004

*CI* confidence interval, *OP* observation point, *RR* relative risk

## Discussion

Our integrated DQMIS resulted in exceptionally high data quality for the trial. Error rates in clinical trials have been reported as high as 26.9%, and could range even higher due to a lack of standardization of data quality measurement [19]. Our overall error rate of 1.67%, as measured by accuracy auditing, provides evidence for the feasibility and effectiveness of integrating DQA into the implementation of health systems research trials. Our DQMIS was successful, despite a steady increase in staff volume, complex and multiple data sources, a vast geographic catchment area across 24 districts, and a large sample size. This success is largely attributable to a number of factors, which we describe below.

### Well-designed technology and data collection processes

It is essential to plan for data quality control mechanisms during the design phase of QI and health systems research trials [31]. We guaranteed completeness, precision, and integrity of data throughout implementation of the trial through several layers of quality control. Stringent and deliberate front-end data entry rules prevented data collectors from entering values outside specified ranges or choosing options that contradicted previous responses. Additionally, significant time and resources were dedicated to implementing robust back-end restrictions into the data collection system to prevent data loss or corruption from occurring. The reporting system enabled the study team, based in India and the US, to monitor data collection indicators to ensure consistent data collection processes. As reported elsewhere [5, 31], this forethought and design facilitated a high-quality dataset.

### Well-defined SOPs

It has also been acknowledged that SOPs and indicator definitions are essential for reliable and accurate data collection in clinical trials [5, 11, 22, 24]. Prior to data collection in the trial, the study protocol was systematically designed with a focus on ensuring data quality through standardization of processes. Data collection tools were designed with validated questions, pre-tested, and finalized through an iterative process. As a reference for data collectors and supervisors, tool guides were developed which included instructions for how to use instruments, definitions, and interpretation guidelines for each question. Tool guides also reinforced consistency of data collection and entry to ensure reliability. Tool guides were adapted and refined throughout the trial to address definitional and other challenges that arose during data collection. Additionally, SOPs and trainings emphasized the importance of timely data entry, reducing the possibility of lost data or inaccuracy as a result of data entry delay.

### Integration into data collection workflow

While methods for assessing data quality in patient registries and health information systems are known, little has been recently published on integrating DQA methods into clinical trial data collection workflows [5, 9, 11–14, 24]. By integrating the DQA protocol into daily workflows, supervisors had the opportunity to support quality throughout implementation of the study. Assigning challenging targets for the intensive phase and lessening these in the maintenance phase reinforced our integrated and continuous system of quality improvement. Following orientation, each data collector was held to high performance standards, fostered by our supportive supervision model. Once achieving intensive phase targets, data collectors were still held to the same targets, but on a less frequent basis to routinely check and bolster accuracy. The aim was to make data collectors accountable for their own performance quality. In addition, the integrated nature of the DQMIS ensured that the proportion of data checked for quality was adapted to the performance of the data collector. The design of the DQA protocol established that the proportion of data checked for quality should be determined by a data collector’s ability to achieve certain performance targets. Target achievement and ongoing supportive supervision together influenced sustained quality throughout implementation. While the ratio of data collectors to supervisors ranged from 2:1 to 4:1 depending on the data collection activity, future trials should consider data collection volume, geographic scope, and minimum quality standards when determining human resource needs for DQA.

### Data feedback paired with supportive supervision

Coaching for QI, when paired with performance monitoring and data feedback, has been shown to be effective in healthcare and other disciplines [32–34]. Recognizing this, we designed a complementary supportive supervision and data feedback model for DQA. Our near real-time reporting system facilitated the continuous monitoring of data accuracy. The design of the system, to rapidly analyze and report on audit results, enabled supervisors to promptly provide support to data collectors to improve data quality. Our supportive supervision model placed an emphasis on building capacity and promoting quality instead of penalizing lower performers. Supervisors were trained in coaching and mentorship techniques in order to emphasize strengths and target areas of improvement. Achievement of accuracy targets was celebrated, and improvement strategies were mutually identified between data collectors and supervisors. The combination of timely data feedback and supportive supervision was integral to the success of the DQA protocol.

### Impact on data collection

During trial implementation, the DQMIS had multiple impacts on data collection methods and refinement of certain questions. Data quality reports highlighted specific concerns related to facility-based observers’ definitional interpretation of key study variables. In one instance, reports demonstrated low accuracy for the observation checklist item: “Was the following available at the bedside: sterile scissors or blade to cut cord.” Supervisors informed managers and study staff of wide variability in data collectors’ interpretation and definitions of sterility. Given this, study management staff chose to revise this checklist item to: “Was the following available at the bedside: clean scissors or blade to cut cord,” along with comprehensive guidelines on how to interpret whether the items were ‘clean.’ ‘Clean’ was defined as sterilized (directly removed from autoclave or boiler) or having no visible marks (dirt, blood, etc.). Data collectors received training on these changes, and scenario-based role playing helped to test their understanding. Following this, subsequent monthly accuracy rates for this checklist item increased to 100% for the duration of implementation. In the absence of data quality reports, inaccurate and unreliable data collection would have persisted.

### Limitations

There are a few limitations to the design and implementation of the DQMIS. First, it is possible that our reliance on the supervisor as the gold standard for delivery observation may have resulted in data incorrectly being considered accurate. There was no other available gold standard, however; therefore, this choice was the most reliable option in the absence of alternatives. Additionally, facility staff not employed by the study entered data in facility registers. For this reason, our DQA is unable to verify the reliability of registration data. We also lack evidence of the cost-effectiveness of the DQMIS. Finally, in order to conduct DQA auditing of facility-based field workers and provide support across the vast geographic size of the study catchment area, the nearly 2:1 ratio of these workers to supervisors was required. This may not be feasible or necessary in other settings.

## Conclusions

The findings of this study demonstrate that integrated methods of DQA combined with SOPs, rapid data feedback, and supportive supervision during trial implementation are feasible, effective, and necessary to ensure high-quality data. In the absence of widely disseminated data quality methods and standards for large health systems RCT interventions, we developed the DQMIS to ensure reliability and serve as a model for future trials. Future efforts should focus on standardization of DQA processes and reporting requirements for data quality in health systems research.

## Abbreviations

DQA: Data Quality Assurance
DQMIS: Data Quality Monitoring and Improvement System
M&E: Monitoring and evaluation
OP: Observation point
QI: Quality improvement
RCT: Randomized controlled trial
SOP: Standard operating procedure

## References

[1] CNS Summit Data Quality Monitoring Workgroup Core Members (2016). Data quality monitoring in clinical trials: has it been worth it? An evaluation and prediction of the future by all stakeholders. Innov Clin Neurosci..

[2] Zozus MNH, Green B, Kahn M, Richesson R, Rusincovitch S, Simon G, Smerek M. Assessing data quality for healthcare systems data used in clinical research (V. 1.0). In Collaboratory phenotypes, data standards, and data quality core. NIH Collaboratory 2014. https://www.nihcollaboratory.org/Products/Assessing-dataquality_V1%200.pdf.

[3] Brown J, Kahn M, Toh S (2013). Data quality assessment for comparative effectiveness research in distributed data networks. Med Care.

[4] Davis JRN, Vivian P, Woodcock J, Estabrook RW (1999). Assuring data quality and validity in clinical trials for regulatory decision making: workshop report.

[5] Nahm ML, Richesson RL (2012). Data quality in clinical research. Clinical research informatics.

[6] Moher D (1998). Does quality of reports of randomised trials affect estimates of intervention efficacy reported in meta-analyses?. Lancet.

[7] Alsumidaie MA, Andrianov A. How do we define clinical trial data quality if no guidelines exist? Applied clinical trials. 2015. http://www.appliedclinicaltrialsonline.com/how-do-we-define-clinical-trial-data-quality-if-no-guidelines-exist.

[8] Goldhill DR, Sumner A (1998). APACHE II, data accuracy and outcome prediction. Anaesthesia.

[9] Richesson RL (2013). Electronic health records based phenotyping in next-generation clinical trials: a perspective from the NIH Health Care Systems Collaboratory. J Am Med Inform Assoc.

[10] Society for Clinical Data Management. Good clinical data management practices. 2013.

[11] Houston L, Probst Y, Humphries A (2015). Measuring data quality through a source data verification audit in a clinical research setting. Stud Health Technol Inform..

[12] Weiskopf NG, Weng C (2013). Methods and dimensions of electronic health record data quality assessment: enabling reuse for clinical research. J Am Med Inform Assoc.

[13] Kahn MG (2012). A pragmatic framework for single-site and multisite data quality assessment in electronic health record-based clinical research. Med Care..

[14] Chen H (2014). A review of data quality assessment methods for public health information systems. Int J Environ Res Public Health.

[15] Nahm ML, Pieper CF, Cunningham MM (2008). Quantifying data quality for clinical trials using electronic data capture. PLoS One.

[16] van der Putten E (1987). A pilot study on the quality of data management in a cancer clinical trial. Control Clin Trials.

[17] Horbar JD, Leahy KA (1995). An assessment of data quality in the Vermont-Oxford Trials Network Database. Control Clin Trials.

[18] Shelby-James TM (2007). Handheld computers for data entry: high tech has its problems too. Trials..

[19] Goldberg SI, Niemierko A, Turchin A (2008). Analysis of data errors in clinical research databases. AMIA Annu Symp Proc..

[20] Hong MKH, Yao HHI, Pedersen JS, et al. Error rates in a clinical data repository: lessons from the transition to electronic data transfer—a descriptive study. BMJ Open. 2013;3:e002406. 10.1136/bmjopen-2012-002406 PMC3657671 23793682

[21] Macefield RC (2013). A systematic review of on-site monitoring methods for health-care randomised controlled trials. Clin Trials.

[22] Arts DGT, de Keizer NF, Scheffer G-J (2002). Defining and improving data quality in medical registries: a literature review, case study, and generic framework. J Am Med Inform Assoc.

[23] Wang RY, Strong DM (1996). Beyond accuracy: what data quality means to data consumers. J Manage Inf Syst.

[24] Gassman JJ (1995). Data quality assurance, monitoring, and reporting. Control Clin Trials.

[25] Richardson D, Chen S (2001). Data quality assurance and quality control measures in large multicenter stroke trials: the African-American Antiplatelet Stroke Prevention Study experience. Trials.

[26] Harvard School of Public Health. BetterBirth: a trial of the WHO safe childbirth checklist program*.* ClinicalTrials.gov [NCT02148952]. National Library of Medicine (US); 2014. https://clinicaltrials.gov/ct2/show/NCT02148952.

[27] Semrau K, Hirschhorn LR, Kodkany B, Spector J, Tuller DE, King G, Lisptiz S, Sharma N, Singh VP, Kumar B, Dhingra-Kumar N, Firestone R, Kumar V, Gawande A (2016). Effectiveness of the WHO safe childbirth checklist program in reducing severe maternal, fetal, and newborn harm: study protocol for a matched-pair, cluster randomized controlled trial in Uttar Pradesh, India. Trials.

[28] Brown W. Data quality assurance tool for program level indicators. MEASURE Evaluation, 2007.

[29] Fitzmaurice GM (2014). Almost efficient estimation of relative risk regression. Biostatistics.

[30] Carter RE, Lipsitz SR, Tilley BC (2005). Quasi-likelihood estimation for relative risk regression models. Biostatistics.

[31] Needham DM (2009). Improving data quality control in quality improvement projects. International J Qual Health Care.

[32] Shojania KG, Grimshaw JM (2005). Evidence-based quality improvement: the state of the science. Health Aff (Millwood).

[33] Hayes E, Kalmakis KA (2007). From the sidelines: coaching as a nurse practitioner strategy for improving health outcomes. J Am Acad Nurse Pract.

[34] Ivers NM (2014). Growing literature, stagnant science? Systematic review, meta-regression and cumulative analysis of audit and feedback interventions in health care. J Gen Intern Med.

